# The myth of the 1-day training: the effectiveness of psychosocial support capacity-building during the Ebola outbreak in West Africa

**DOI:** 10.1017/gmh.2019.2

**Published:** 2019-05-07

**Authors:** Rebecca Horn, Fiona O'May, Rebecca Esliker, Wilfred Gwaikolo, Lise Woensdregt, Leontien Ruttenberg, Alastair Ager

**Affiliations:** 1 Institute for Global Health and Development, Queen Margaret University, Edinburgh, UK; 2 University of Makeni, Makeni, Sierra Leone; 3 LiCORMH, Monrovia, Liberia; 4 War Trauma Foundation, Amsterdam, Netherlands

**Keywords:** Capacity building, Ebola, liberia, mental health, psychological first aid, psychosocial support, Sierra Leone

## Abstract

**Background.:**

In emergencies and resource-poor settings, non-specialists are increasingly being trained to provide psychosocial support to people in distress, with Psychological First Aid (PFA) one of the most widely-used approaches. This paper considers the effectiveness of short training programmes to equip volunteers to provide psychosocial support in emergencies, focusing particularly on whether the PFA training provided during the Ebola outbreak enabled non-specialists to incorporate the key principles into their practice.

**Methods.:**

Semi-structured interviews were conducted in Sierra Leone and Liberia with 24 PFA trainers; 36 individuals who participated in PFA training; and 12 key informants involved in planning and implementing the PFA roll-out.

**Results.:**

Findings indicate that many PFA training-of-trainers were short and rarely included content designed to develop training skills. As a result, the PFA training delivered was of variable quality. PFA providers had a good understanding of active listening, but responses to a person in distress were less consistent with the guidance in the PFA training or with the principles of effective interventions outlined by Hobfoll *et al*.

**Conclusions.:**

There are advantages to training non-specialists to provide psychosocial support during emergencies, and PFA has all the elements of an effective approach. However, the very short training programmes which have been used to train non-specialists in PFA might be appropriate for participants who already bring a set of relevant skills to the training, but for others it is insufficient. Government/NGO standardisation of PFA training and integration in national emergency response structures and systems could strengthen in-country capacity.

## Introduction

In emergencies and resource-poor settings across the world, non-specialists (people who lack prior professional or other specialised training in mental health and/or psychosocial support) are increasingly being trained to provide emotional and psychosocial support to people in distress (Mendenhall *et al*., 2014; Haruf *et al*., 2015; McLean *et al*., 2015; O'Hanlon & Budosan, 2015; Singla *et al*., 2017). These people may include community health volunteers, peer helpers, midwives, auxiliary health staff, teachers and those without a professional service role but who are active and respected within their communities, such as religious leaders and community leaders (McLean *et al*., 2015).

The training-of-trainer (ToT) model is widely used to build mental health and psychosocial support (MHPSS) capacity in humanitarian situations (Baron, 2006). This can be successful but also has disadvantages. There is a risk of the material becoming diluted or misrepresented as successive groups of trainers provide trainings, and ‘in a field as varied, dynamic, and nuanced as mental health (and psychosocial support), certain concepts are bound to be lost through misunderstanding or the chosen focus of a particular trainer, and when that trainer has not been exposed to a full education in mental health (and psychosocial support), some key ideas or approaches may go missing’ (Haruf *et al*., 2015: 15).

### Psychological first aid

An example of an approach to MHPSS in emergencies which makes significant use of non-specialists is Psychological First Aid (PFA). Although the term has been used since the 1940s, PFA in its current, most widely used form emerged in 2011, when the World Health Organisation, War Trauma Foundation, and World Vision published a PFA guide for fieldworkers and accompanying training materials. It aims to facilitate recovery in the aftermath of a disaster or crisis by reducing initial distress, helping affected individuals to meet their basic needs and connect with social supports and services, providing information, and fostering short- and long-term adaptive functioning and coping.

This version of PFA is based on a systematic review commissioned by WHO (Bisson & Lewis, 2009), which showed that there was little evidence for some of the most commonly-used approaches to supporting people who had experienced very distressing events, and, indeed, that some widely used approaches could be harmful. Although the review found an absence of direct evidence for formal interventions to support those involved in a traumatic event, they were able to identify some characteristics of good interventions, drawing in particular on two resources. One was a Delphi study conducted to develop the European Network for Traumatic Stress's (TENTS) Guidelines on psychosocial care following disasters (Bisson & Tavakoly, 2008). The second was a paper by Hobfoll *et al*. (2007), which reports on the conclusions of a panel of international experts on the study and treatment of those exposed to disaster and mass violence. Hobfoll *et al*. put forward five general principles for successful interventions or policies, which were supported by the empirical literature and which the authors termed ‘evidence – informed.’ These principles underpin the PFA approach, as outlined in Table 1.

**Table 1.** Five principles of effective interventions (Hobfoll et al., 2007) and their relationship with PFA approach (WHO, War Trauma Foundation, World Vision manual, 2011)

Principle	Description	Integration into PFA approach
Promote sense of safety	Ensure that the affected person is in a safe place and has the information they need to keep themselves safe, including clear and accurate information about the situation. Information about the welfare of relatives is also important.	Creating and maintaining a safe environment is a theme throughout the PFA approach in terms of physical safety, ensuring that the person feels safe and comfortable during their interaction with the PFA provider and that they have access to relevant information.
Promote calming	Prolonged anxiety can lead to further psychological and physical problems, and have negative effects on decision-making, interpersonal relationships, and daily functioning. An effective approach to people in distress includes helping the individual to develop the skills to achieve a relaxed state (e.g. breathing exercises) and providing accurate information about normal reactions to distressing events. Effective interventions also include facilitating problem-focused coping, which involves assisting and guiding individuals to break down a problem into small, manageable units. This not only reduces problems but also increases a sense of control (promoting self-efficacy) and provides opportunities for small achievements (promoting hope).	The PFA approach includes ‘comforting people and helping them to feel calm’ (p3) through staying close to them, not pressuring them to talk, listening if they do want to talk, and using various calming techniques such as encouraging them to focus on their breathing. It also includes helping people to identify and prioritise their most urgent needs.
Promote sense of self- and collective efficacy	The sense that one can cope with events has been found to be beneficial for recovery after distressing experiences, so a key element of an effective approach is a focus on imparting skills to the individual and/or reminding them of their ability to cope with difficulties. Self-efficacy does not imply that the individual solves their problems alone, rather that they are able to link into resources both within themselves and outside themselves in order to address their concerns. People need to be linked to external resources in order to meet their needs but also require the skills to meet their goals and the feeling that they have the capacity to take action in the future. The related skills, beliefs, and resources mutually influence each other.	The PFA approach emphasises the importance of ‘help[ing] people to help themselves and to regain control of their situation’ (p24). It includes a focus on helping people to identify and use positive coping strategies that have benefited them in the past and identifying existing supports in their lives.
Promote connectedness	There is a compelling body of evidence on the central importance of social support and sustained attachments to loved ones and social groups in combating stress and strengthening psychosocial wellbeing. Good social networks enable information to be communicated, plus a range of social support activities to take place including practical problem solving, emotional support and sharing experienced and helpful coping strategies. ‘This, in turn, can lead to a sense of community efficacy’ (Hobfoll et al., 2007: 296).	Connecting with loved ones and social supports is a key element of the PFA approach. Suggested actions include ensuring that families are kept together; helping people connect with friends and relatives; connecting people with their spiritual community; and bringing affected people together to help each other.
Promote hope	There is strong evidence for the importance of retaining hope after distressing events, with those who are able to do so showing more positive outcomes. Hope can be promoted at an individual level by focusing on people's strengths, by psychoeducation which normalises people's responses, and by encouraging positive coping behaviours.	Whilst promoting hope is not explicitly part of the PFA approach, a focus on people's strengths and ability to cope with difficulties is a theme which runs throughout the guidance. There is also a section on spirituality, which notes how faith and religion can give a sense of hope in the midst of a crisis.

Since 2011, the PFA approach has been widely adopted by organisations and government bodies responding to emergencies (Church of Sweden, 2018). WHO and others have made comprehensive training materials available and advise that the training can be delivered in 1 day to anybody who is in a position to offer early assistance to affected individuals. Currently, there has been no systematic study of the PFA approach in emergency settings. There is a general acknowledgement of a need to learn more about its effectiveness in such settings (e.g. Schulz & Forbes, 2013; Church of Sweden, 2018), and build the evidence base for the practical application of PFA.

### The Ebola outbreak in Sierra Leone and Liberia

In 2014 an outbreak of the Ebola Virus Disease (EVD) began in a number of West African countries, including Sierra Leone and Liberia. Ebola is a highly contagious virus, with an extremely high mortality rate. As of April 2016, Sierra Leone had reported 14 124 cases of suspected, probable and confirmed EVD and 3956 deaths; Liberia had reported 10 678 cases and 4810 deaths (CDC, 2016).

PFA formed a central part of the psychosocial response to the EVD outbreak in both Sierra Leone and Liberia. WHO *et al*. (2014) produced an adapted version of the PFA Guide and training materials specifically for use with populations affected by Ebola. A wide range of people was trained to providing psychosocial support using the PFA approach, including health care workers, community leaders, teachers, and social workers.

The aim of the study reported here is to understand how the PFA approach was used during the Ebola outbreak in Sierra Leone and Liberia, and to learn lessons from this which can be applied in other emergency contexts to strengthen the psychosocial support offered by first responders.

## Methodology

In each country, data collection was led by a research coordinator (third and fourth authors) who recruited the research assistants locally. In Sierra Leone, four research assistants were recruited who both conducted and transcribed the interviews. In Liberia, four research assistants were recruited to conduct interviews, plus four transcribers. The data collection process was supported by the lead researcher (first author) who trained the research assistants and transcribers, and provided additional supervision and support to research coordinators, research assistants and transcribers during the first week of data collection. The lead researcher and the research coordinators conducted the key informant interviews.

Individual interviews were conducted with:

- Individuals who had been trained as PFA trainers during the EVD outbreak and had subsequently delivered PFA training to others (referred to as ‘trainers’).
- Individuals who had participated in PFA training during the EVD outbreak and had subsequently used the PFA approach in their work (referred to as ‘providers’).
- Individuals who played a role in planning and implementing the roll-out of PFA as part of the overall EVD response in Sierra Leone or Liberia (referred to as ‘key informants’).

### Recruitment

In both countries, participants were selected through a purposive sampling technique to represent key strata of those trained to provide PFA training during the acute and early recovery stages of the emergency. Organisational representation, gender, age, urban-rural settings, and professional background were considered during the selection process. The inclusion criteria were:

- Received TOT training in PFA between 1 April 2014 and 31 March 2016 in Liberia
- Provided PFA training to other stakeholders during this time
- A formally recognised PFA trainer

In Sierra Leone, trainers were recruited using an existing 4Ws Mapping document compiled by IASC (2012). The Sierra Leone research coordinator and research assistant contacted all agencies that had provided PFA ToTs during the period of interest. Those who responded were provided with more detailed information regarding the interviews and invited to participate.

In Liberia, mental health supervisors in each county supported the selection of participants. Once a potential participant was identified, he/she was contacted by trained data collectors to verify his/her participation in PFA training. He/she was then provided with information about the study and invited to take part. If he/she agreed, two research assistants sat with him/her to conduct the interview.

The selection of key informants focused on those people who were involved in the MHPSS element of the EVD response in Sierra Leone and Liberia, so had specialist knowledge relating to the roll-out of PFA in that context. Unfortunately, many of the key individuals had since left West Africa and could not be interviewed. Interviews were conducted September–October 2016.

### Interviews with PFA trainers

Semi-structured interviews were conducted with 23 trainers (15 in Sierra Leone and 8 in Liberia; see Table 2 for demographic details). These interviews explored how PFA training was delivered during the EVD crisis, whether fidelity to the original model was maintained, and the trainers' reflections on the process of rolling out the training (Appendix 1).

**Table 2.** Description of respondents

Id	Country	Location	Gender	Sector	Role/Position at time of PFA training	Setting	Years in practice
**Trainers**							
SL_T_01	Sierra Leone	Freetown	F	Health	SRN/Mental health nurse	Urban	8
SL_T_02	Sierra Leone	Freetown	M	Health	Child protection manager	Urban	10
SL_T_03	Sierra Leone	Freetown	F	Health	Mental health nurse	Urban	8
SL_T_04	Sierra Leone	Freetown	F	Health	Administrator	Mix urban/rural	4
SL_T_05	Sierra Leone	Freetown	M	Health	Mental health nurse	Urban	3
SL_T_06	Sierra Leone	Freetown	M	Health	Mental health nurse	Rural	3
SL_T_07	Sierra Leone	Freetown	M	Health	Professional counsellor	Urban	26
SL_T_08	Sierra Leone	Makeni	M	Health	Community health coach	Mix urban/rural	6
SL_T_09	Sierra Leone	Makeni	M	Social work	Senior Social Officer	Urban	2
SL_T_10	Sierra Leone	Makeni	F	Education	Volunteer provider PFA	Urban	n/a
SL_T_11	Sierra Leone	Kenema	F	Health	Mental health nurse	Urban	2
SL_T_12	Sierra Leone	Bo	F	Health	Safe and Dignified Burial Team	Mix urban/rural	1
SL_T_13	Sierra Leone	Bo	F	Health	Safe and Dignified Burial Team	Mix urban/rural	2
SL_T_14	Sierra Leone	Bo	F	Health	Safe and Dignified Burial Team	Urban	15
SL_T_15	Sierra Leone	Bo	M	Social work	Regional Disaster Officer	Urban	2
L_T_01	Liberia	Montserrado County	F	Health	Mental health Nurse	Urban	4
L_T_04	Liberia	Montserrado County	F	Health	Mental health clinician	Urban	11
L_T_06	Liberia	Montserrado County	M	Health	Mental health clinician	Urban	3
L_T_07	Liberia	Montserrado County	F	Community	Community volunteer	Urban	2
L_T_08	Liberia	Grand Cape Mount County	F	Health	Nurse	Rural	9
L_T_09	Liberia	Margibi County	F	Health	Mental Health Supervisor/Nurse	Urban	4
L_T_11	Liberia	Montserrado County	M	Health	Psychosocial Focal Point for MOH	Urban	7
L_T_12	Liberia	Montserrado County	M	Health	Nurse/MHC	Mix urban/rural	4
**PROVIDERS**							
SL_P_01	Sierra Leone	Freetown	M	Social work	Social worker	Urban	2
SL_P_02	Sierra Leone	Freetown	M	Health	Clinical Health Officer	Rural	8
SL_P_03	Sierra Leone	Freetown	M	Social work	Child Protection Officer	Rural	5
SL_P_04	Sierra Leone	Freetown	M	Social work	Social worker	Urban	3
SL_P_08	Sierra Leone	Makeni	M	Social work	Social worker	Urban	5
SL_P_09	Sierra Leone	Makeni	M	Education	Volunteer social worker	Urban	12
SL_P_11	Sierra Leone	Kenema	M	Health	Lab technician	Unknown	Unknown
SL_P_12	Sierra Leone	Kenema	F	Health	Lab technician	Urban	Unknown
SL_P_13	Sierra Leone	Kenema	M	Social work	Social mobiliser	Mix urban/rural	2
SL_P_14	Sierra Leone	Kenema	M	Social work	Volunteer social worker	Urban	10
SL_P_15	Sierra Leone	Kenema	M	Community	Rural Chief Ebola Task Force	Rural	2
SL_P_16	Sierra Leone	Kenema	F	Health	Social mobiliser	Rural	5
SL_P_18	Sierra Leone	Kenema	M	Community	DHMT PSS team	Unknown	2
SL_P_19	Sierra Leone	Bo	M	Health	Safe and Dignified Burials	Mix urban/rural	1
SL_P_20	Sierra Leone	Bo	M	Health	Safe and Dignified Burials	Urban	3
SL_P_21	Sierra Leone	Bo	M	Social work	Regional Child Justice Officer	Mix urban/rural	2
SL_P_22	Sierra Leone	Kenema	M	Social work	Anti Human Trafficking Officer	Urban	3
L_P_02	Liberia	Margibi	F	Education	Teacher	Urban	9
L_P_03	Liberia	Montserrado County	F	Health	Social Worker	Mix urban/rural	2
L_P_04	Liberia	Montserrado County	M	Health	Social Worker	Urban	6
L_P_05	Liberia	Margibi County	M	Community	Community Leader	Urban	2
L_P_06	Liberia	Margibi County	M	Education	Teacher	Urban	16
L_P_07	Liberia	Grand Cape Mount Co.	M	Education	Teacher	Urban	10
L_P_08	Liberia	Margibi County	F	Health	Nurse/RN	Rural	3
L_P_09	Liberia	Margibi County	F	Health	Midwife	Urban	2
L_P_10	Liberia	Grand Cape Mount	M	Health	Nurse	Rural	4
L_P_11	Liberia	Montserrado County	F	Education	Care Giver	Rural	5
L_P_13	Liberia	Montserrado County	F	Health	Social Worker	Mix urban/rural	6
L_P_14	Liberia	Margibi County	M	Education	Teacher	Urban	5
L_P_15	Liberia	Margibi County	M	Education	Teacher	Urban	3
L_P_16	Liberia	Grand Cape Mount Co.	F	Education	Teacher	Rural	15
L_P_17	Liberia	Grand Cape Mount Co.	M	Education	Teacher	Rural	10
L_P_18	Liberia	Montserrado County	F	Health	Nurse/MHC	Urban	8
L_P_19	Liberia	Margibi County	F	Health	Nurse	Rural	5
L_P_20	Liberia	Grand Cape Mount Co.	F	Health	PA	Rural	1
L_P_21	Liberia	Montserrado County	F	Social Work	Social Worker	Urban	5

### Interviews with PFA providers

Interviews were conducted with 36 providers (17 in Sierra Leone and 19 in Liberia, see Table 2) to explore how the PFA approach was understood and used by those who were trained (Appendix 2). Interviews with both trainers and providers were conducted in either English or an agreed local language.

All interviews were audio recorded, with the permission of participants.

### Interviews with key informants

Interviews were conducted in English with 14 key informants, and audio recorded (six in Sierra Leone and eight in Liberia), in order to understand how PFA was rolled out during the EVD outbreak in Sierra Leone and Liberia, including the perceived strengths of the approach, the challenges experienced, and ways in which the PFA approach was adapted for the context.

Five key informants (three female, two male) were interviewed in Sierra Leone, plus one interview was conducted through Skype with a key informant (female) who is now working outside Sierra Leone. Four key informants for Sierra Leone worked for government Ministries at the time of the EVD outbreak and two worked for the World Health Organisation (WHO).

Six key informant interviews were conducted in Liberia, two of which engaged two participants (two male and six female). Four key informants in Liberia worked for government Ministries at the time of the EVD outbreak; two for WHO and two for UNICEF.

### Analysis

The voice recordings were transcribed in English, and the transcripts then checked against the voice recording. The first author developed a coding scheme involving three levels with which excerpts of the data could be labelled. The first was based on the broad themes around which the interview questions were organised (e.g. provider reflections on PFA training). The second level focused on issues covered within each theme (e.g. within ‘provider reflections on PFA training’ the second level codes included provider training needs; selection process; training content and methods; supervision; refresher training). The third level related to the content of the interview data on each particular issue (e.g. within ‘provider reflections on PFA training/supervision’ the third level codes were ‘supervision positive’, ‘supervision negative’ and ‘supervision recommendations’). The lead researcher then worked with two other analysts (second and fifth authors) to trial the coding scheme, discuss discrepancies, and make modifications. The final round of testing indicated a high degree of agreement among coders (intraclass correlation  = 0.80 or above), following which all three coders worked on coding the transcripts using the on-line analysis package, Dedoose. Once the coding was complete, the same team worked on the data analysis. The draft results were shared with and discussed by the entire research team at a workshop in January 2017, following which they were revised and finalised.

### Ethics

This study was given favourable ethical approval by Queen Margaret University Edinburgh Research Ethics Committee, the Office of the Sierra Leone Ethics and Scientific Review Committee, and the University of Liberia Pacific Institute for Research Review Board (UL-PIRE).

## Results

### Cultural adaptation of the PFA approach

As noted above, the PFA facilitators' guide was adapted for the Ebola situation (WHO *et al.*, 2014). In Liberia, this manual was used by all the organisations whose representatives were spoken to for this study. The content itself was perceived to be appropriate and not to require adaptation. Trainers said the only modifications they made to the material were in terms of language and adapting the role plays and other exercises to be suitable for the group being trained. Although the original manual was used to deliver PFA training, additional emphasis was given to safe entry into communities, self-care, and active listening skills. These elements were felt to be especially important in the Liberia context during the EVD outbreak.

In Sierra Leone, there was a difference between the training delivered under the mandate of the Ministry of Health and that delivered under the mandate of the Ministry of Social Welfare, Gender and Children's Affairs. The PFA training delivered by organisations associated with the Ministry of Health was based primarily on the PFA manual (EVD version) but was integrated into a broader training programme. The other issues incorporated into this training programme included stress management, and medical issues related to EVD (e.g. the management procedure). The Ministry of Health felt that the PFA manual (EVD version) was appropriate in terms of content and did not make any adaptations apart from wording and language. Organisations and agencies working in partnership with the Ministry of Social Welfare, Gender and Children's Affairs used an adapted training manual, which included a 95-min session on PFA. The PFA element of the training package was based closely on the WHO *et al*. (2014) manual but was greatly reduced to fit into the 95-min time slot. The emphasis on safe entry into the community, active listening, and self-care which was evident in Liberia, was also present in the PFA trainings delivered in Sierra Leone.

There was agreement amongst respondents that the PFA manual (EVD version) was appropriate and did not require adaptation in terms of its general content. However, several noted the need to ensure that the training was culturally appropriate in terms of community entry, how to approach a distressed individual, and in the language and case studies used.

*‘We talked about the respect of the culture, we talked about how it influences our behaviour, we talked about how it prevents other positive things from happening; we talked about how we as a group can adopt a positive behaviour in addressing some of those issues that may not be hurtful to that culture'.* (Trainer, Liberia)

### Rolling out PFA training during the EVD outbreak

In both Sierra Leone and Liberia there was some delay in identifying the EVD outbreak as an ‘emergency'. This resulted in confusion over how to organise the response, and where psychosocial support and PFA fitted in. As the EVD crisis developed, many new organisations arrived and began providing services, creating challenges for coordination and making it difficult to control the quality of the trainings being delivered. There were instances of poor-quality trainings being offered, and of the same people being trained multiple times by different organisations.

*‘It was diluted, people would do different things and call it PFA, and that was not PFA … Later on a lot of other organisations started training, we don*'*t know what methods they were using, somebody would say ‘we are going to use PFA’, they would do two hours, three hours*’ (Key informant, Liberia).

It was not always possible to find people with existing skills in psychosocial support who could be trained as PFA trainers, and the ToTs were shorter than would ideally be the case because it was difficult to take people out of the work environment for long. The limited training time had an impact on quality. For example, some short ToTs did not include any content on how to plan and deliver a training session. There was considerable variation in whether trainers received supervision as they delivered their first PFA training courses to others, or refresher training after they had started to train others.

Most of the PFA providers interviewed had been selected for training because their role involved contact with distressed individuals, but some were selected because they were working in very distressing situations and were in need of emotional support themselves. In the absence of any kind of stress management programmes, they were selected for PFA training to help them learn ways to cope with the situation they were working in.

Most providers said they received some form of supervision after they participated in the PFA training, and around half said they participated in refresher training, but the nature and quality of both the supervision and refresher training varied considerably. The providers who had not received supervision would have welcomed it, and trainers also felt that it was necessary.

‘*This will help everybody to know where the gaps are because if you have been trained and are not being supervised, you just continue to go you think that all is well. Probably there might be a gap you don*'*t know and if you would have been supervised the gap will be filled'* (Trainer, Liberia)

*‘If you only come and train me today and you go, never to come and monitor what I'm doing, whether it*'*s right or not, it means that your training is in vain*’ (Provider, Liberia).

### Effectiveness of the PFA training

The data are discussed here in relation to Hobfoll *et al*.’s (2007) five key principles. These key principles relate essentially to the ‘responding’ part of the PFA approach, rather than the information-gathering elements (preparing, observing, listening). Yet, it is important to note that the ‘listening’ element of the PFA approach was perceived as particularly important when responding to a person in distress. Most providers were clear on the elements of active listening, such as not interrupting, allowing the person to tell their story in their own way, and showing through non-verbal behaviour and minimal responses that one is paying attention.

‘*We had been taught that the person opens up to you to the extent that you provide the encouragement. You should not direct the discussion to tell the person to, for example, skip to the end of the story. No. Even though you might come in occasionally but you can only come in relation to what the person has already told you. That would give the person the encouraging thought to continue’* (Provider, Sierra Leone).

However, there was less consistency with the guidance in the PFA training when it came to the way in which providers said they responded to people in distress, as discussed below.

#### Promote sense of safety

There was a clear focus on safety, due to the EVD context in which responders were very aware of the life-or-death situation they were working in. PFA providers understood the need to ensure their own safety and that of the people they were supporting and would do so both through practical means and by providing information.

#### Promote calming

The ‘calming’ element of the PFA approach was described as particularly important during the EVD outbreak since people paralysed by fear and panic tended to make bad decisions and potentially expose themselves and others to EVD.

*‘Psychological first aid, and psychosocial support in general was the crux of helping people to overcome the fear, which is what was stopping people from making good health decisions. So we were sent in when families refused to stay under observation. We were sent in when families refused to give up someone, they were hiding someone who was a contact’* (Key informant, Liberia).

Our data show that the PFA providers in Sierra Leone and Liberia recognised the need to calm the individuals they were supporting. However, they rarely described the approaches recommended in the PFA training, such as grounding techniques, relaxation techniques, or helping the individual to break down problems into small units and address these issues one by one (which also helps to promote self-efficacy). More commonly, they tried to calm people down by reassuring them, telling them that things would be fine, and by giving stories of other individuals who have experienced similar situations.

*‘We talk to him that what happened to him wasn*'*t nobody*'*s doing but God so let him have the faith that it*'*s Gods doing, we at times cherish things which are not approved by God so let him exercise patience and look up to God, He will give him another good wife that can bear him other children’* (Provider, Sierra Leone).

*‘You tell the person that, ‘My friend you are not the only one facing stigma. I am facing stigma too. Maybe mine is even worse than yours.’ He would say, ‘No, mine is worse.’ Then you will be able to explain your situation to him. You can then come up with a scenario not necessarily true just to cajole him and even add that you faced a worse issue than he did. In my case I told him the issues I faced at the workplace, in the home and even in the community. That way I was able to convince him’* (Provider, Sierra Leone).

Hobfoll *et al*. (2007) note that the use of ‘spinning’ information in order to calm an individual ultimately undermines the credibility and is counter-productive (p. 292). The PFA providers interviewed frequently gave advice, or took action themselves to solve the person's problem, and whilst this may have a short-term positive effect in terms of reducing anxiety it does not promote self-efficacy, as discussed below.

#### Promote sense of self – and collective efficacy

This was particularly challenging for PFA providers. It involves helping a distressed individual to recognise their own strengths and resources and connecting them with external supports in a way which enables them to both develop the sense that they have the capacity to address the challenges they face and develop the skills necessary to meet their goals. This requires considerable skill on the part of the provider; if those skills are lacking it is easier to either give advice and/or connect the individual to another source of help. This is, in fact, what was observed in the descriptions of PFA given by those interviewed for this study. The respondents focused on ensuring that the individuals they worked with had access to the external resources they needed, and on making referrals to organisations which could support them further.

The PFA approach does include a focus on helping people to address basic needs and access services, and the PFA providers did this well in the EVD context. However, there was no emphasis in their descriptions on helping people to cope with their problems or on helping people to identify their own resources, strengths, and skills. The providers tended to give advice about the course of action to take, rather than support the person to make their own decisions about the best way forward.

*‘*[*The woman I was helping said*] *I will carry him to court, we will go in the court, in fact … me and him we going get divorce. I say, don*'*t do that, I say when you do that what will happen when you leave the man? This man that you leaving, so you go to another one it will be worse than the other one, you don*'*t know the places you will be with the children, you don*'*t know where you will be tomorrow and also you have to think about your health, how all of them will come up, you and the baby’* (Provider, Liberia).

#### Promote connectedness

Promoting connectedness was challenging in the Ebola context since in order to stay safe people could not meet in groups, and there was fear of social interactions. This was reflected in the respondents' descriptions of supporting people in distress, which focused primarily on linking them to organisations with little emphasis on strengthening relationships or promoting connectedness.

A small number of respondents did describe helping to strengthen connections between distressed persons and their community, church or family members.

*‘After we got the PFA training then, it then even helped us decide the community entry that we were doing, if we had any churches there, and maybe other organisations there that are found within those same community, we will go to them and try to link the families that were involved’* (Provider, Liberia).

One respondent (a religious leader) described how he and some colleagues established a Survivors' Organisation, to strengthen relationships and empower survivors who were previously marginalised within communities. This is an example not only of promoting connectedness, but also promoting a sense of self-efficacy and collective efficacy.

#### Promote hope

PFA providers recognised the central role that hope plays in recovery, and frequently described offering encouragement and giving hope to people in distress. However, many of the examples given came close to offering reassurance and making false promises (e.g. everything will be fine), which are not advocated by the PFA approach, and can, in fact, be harmful. Most respondents saw promising that things will get better as an essential aspect of offering emotional support.

‘*There are things that might happen that makes a person think that it is the end of everything. You want the person to know that she or he should not see it as the end'* (Provider, Sierra Leone).

### Factors contributing to effectiveness of PFA capacity-building

As already described, the difficulties in coordinating PFA training activities during the EVD outbreak made it challenging to control their quality, and they were often very short because people could not be removed from the response for long.

Interviews indicated that in some cases, supportive communication skills were not fully developed before people began offering PFA, and in others there was no clear grasp of the PFA approach itself. Some key informants described a challenge of ensuring that those trained in PFA understood the limits of what they were able to offer and did not start presenting themselves as ‘counsellors’ or ‘psychotherapists’.

Our interviews with providers indicated that PFA was most appropriately used in acute crisis situations (e.g. when someone had lost family members to EVD), whilst a distorted version of PFA was more often used when people were responding to ongoing problematic situations. These examples often indicated a lack of understanding of the purpose and limitations of the approach, and perhaps also a lack of other skills to deal with difficult situations, in particular conflicts between individuals or groups.

‘*There was this time when a woman died who was a leader in the Bondo society. The women insisted on burying her in their traditional fashion. They attempted to do it. We intervened and I called my chairman and we went there and emphasised that even though this was our common tradition yet the existing laws at the time forbade that. So we told them and even though it was still challenging but in the end we prevailed and they complied'* (Provider, Sierra Leone).

Supervision was extremely important, given the short PFA trainings offered and the low capacity of many of those who participated in the trainings, *‘If there was something that I left out, he would come in and contribute some information and we will talk on it a little bit and continue afterwards’* (Trainer, Sierra Leone). Where supervision was not possible, the PFA approach became diluted and confused.

*‘Places where we were not able to supervise, we heard absolutely different things and different ideas, which did not help the people. Actually, that was bad, yeah, that was not good’* (Key informant, Liberia).

*‘Wherever you go they say a lot of trainings have been done …. But you find out that no supervision has been done nor evaluation has been done … some people come in and say they are doing PFA but … you ask a few questions and they cannot even understand and yet they say they have done PFA. So you realise the quality of PFA has been diluted because of lack of supervision, and lack of proper monitoring and evaluation of the process*' (Key informant, Sierra Leone).

## Discussion

There is a general consensus that PFA provides a useful early intervention for crisis-affected people, alongside other essential mental health and psychosocial activities. In this paper, we have explored the ways in which PFA capacity was developed amongst non-specialists during the EVD outbreak in Sierra Leone and Liberia, and the ways in which the approach was used subsequently. Our findings suggest that PFA is not the easily acquired framework which it has been presented as. There is an expectation that participants can acquire core empathic and supportive skills in a short time, and integrate these effectively with their existing attitudes, skills, and knowledge. There is also an expectation that they will use their cultural understanding to make sense of the new approach and assimilate it into their cultural context. At the same time, they are expected to recognise that there are certain elements of the PFA approach which should not be changed because these are consistent with evidence about effective support for people who are distressed (e.g. the Hobfoll *et al*. principles).

Our findings indicate that many of the ToTs in PFA during the EVD outbreak in Sierra Leone and Liberia did not meet the standards outlined by Baron (2006). Respondents typically had little prior experience of MHPSS interventions and no experience of PFA. The ToTs were often short and rarely included content designed to develop training skills. As Baron notes, ‘Trainers have nothing to train if they have not mastered something first’ (p. 111). The process of hearing and understanding new information – which may conflict with existing beliefs, attitudes, and behaviours – takes time, and additional time is needed to integrate this new material into a personal frame of understanding in order to teach it to someone else. ‘It becomes even more complicated when teaching someone how to do something new and then expecting him or her to teach the skill to someone else’ (Baron, 2006: 112). As a result, the PFA training delivered during the EVD outbreak was of variable quality. It has been noted by others that fidelity to the original PFA model has not always been maintained as it is scaled up and mainstreamed in emergency settings (Church of Sweden, 2018).

PFA providers often described using only active listening when they talked about using the PFA approach. They had a good understanding of this element of the approach, but descriptions of actual responses to a person in distress were less consistent with the guidance in the PFA training or with the principles of effective interventions outlined by Hobfoll *et al*. (2007). It is likely to be difficult, or impossible, to change existing, life-long patterns of responding, which may be in line with cultural and social norms, in a 1-day training.

An empathic approach is central to PFA, and the assumption is that either most trainees already have this, or it can be developed relatively easily and quickly. When people combine new skills with existing attitudes and beliefs, without having an empathic approach, there is a danger they could become involved in situations they are ill-equipped to handle, and potentially do harm. For example, in Sierra Leone, it is common (and perhaps expected) that one would comfort a distressed person by promising that everything will be fine. This use of false reassurance ‘ultimately undermines the credibility and is counter-productive’ (Hobfoll *et al*., 2007: 292). Similarly, an attempt to solve a person's problem for them instead of promoting the sense that they have the capacity to cope with distressing events can undermine self-efficacy and ultimately hinder recovery. If a helper is able to empathise with a distressed person, it is possible during training for them to put themselves in the position of that person and understand how these normal responses can potentially be harmful. However, if they learn the new approach from the ‘helper’ perspective only, without being able to empathise with those being helped, then their existing responses may be less likely to change through a short training. This raises the possibility that they will perceive themselves as being more competent than they are and may attempt to help in situations which are beyond their capacities, potentially doing harm.

Empathic responses are influenced by many factors (Brunero *et al.*, 2010), but there is evidence that empathy can be taught to some extent (Teding van Berkhout & Malouff, 2016). Studies of empathy training indicate that its effectiveness depends on it being truly experiential and involving all four components of behavioural skills training (instruction, modelling, practice, and feedback) (Teding van Berkhout & Malouff, 2016). This means, for example, that every participant must both take part in a role-play and receive feedback on their performance since this is where new concept formation and learning is achieved. Simply watching other participants take part in a role-play or demonstration, or participating in a role-play but not receiving personalised feedback, is likely to be less effective. However, when the time is short and numbers are large, as is often the case during an emergency, this is commonly what occurs. McLean *et al*. (2015) note that group training in the MHPSS sector often facilitates knowledge gain but is less effective in promoting skill building, even when role play and other participatory activities are included [also (e.g. Beidas & Kendall, 2010; Budosan, 2011)]. A more effective approach, they found, was coaching individuals within the community, which offers greater opportunities for practice and feedback. Post-training supervision is an essential element of a successful capacity building approach; Fixsen *et al*. (2005) report that post-training supervision is one of the strongest predictors of behaviour change.

Some have noted that low-cost, widely scalable MHPSS interventions are sometimes ‘valued over other interventions that are more resource intensive, but necessary to appropriately care for people in the situation’ (Church of Sweden, 2018: 74). There are clear advantages to training non-specialists to provide psychosocial support during emergencies, and PFA, as outlined in manuals and training materials, has all the elements of an effective approach. However, the perception that it is a cheap and easy option has led to very short training programmes, with minimal follow-up support. The findings of this study indicate that a 1-day PFA training is unlikely to be sufficient for most non-specialists to provide effective, non-harmful psychosocial support, although it may be appropriate for people who already have good communication skills and high levels of empathy.

Participants in psychosocial training are often expected to change their attitudes and beliefs, as well as learn new skills, and the effort involved in this should not be underestimated (Baron, 2006). For those who are entirely new to the field, training on specific psychosocial approaches should be preceded by a general training on supportive communication skills. Psychosocial skills should be taught in groups small enough for every participant to have the opportunity to both practice the skills and receive feedback, and supervision and refresher training should be recognised as an essential part of the process. It is crucial to ensure the quality of ToTs, in the way outlined by Baron (2006). In addition, efforts could usefully be made to contextualise the PFA training materials so that they are more appropriate for the setting in which they are to be used. In 2017 the Sierra Leone WHO mental health specialist, on behalf of the Ministry of Health and Sanitation, developed a PFA ToT manual which remained faithful to the PFA model but brought in additional elements necessary for health care providers in Sierra Leone to use the approach effectively. In emergencies, it can be challenging to make these adaptations, but a more context-specific PFA training is likely to lead to enhanced understanding and more effective use of the approach. These recommendations are in line with those put forward in a recent review of PFA (Church of Sweden, 2018), which notes a need to continue to develop innovative PFA resources and technologies, and to promote fidelity to the model with support to capacity building initiatives and dialogue among communities of practice, as well as development of guidance and support materials for both PFA ToTs and PFA orientations/trainings.

Whilst the training standards outlined here may be hard to maintain in emergency settings, this study indicates that attempting to offer psychosocial support through non-specialists without adequately investing in their learning is not likely to lead to the type of good, safe emotional and practical support we would hope to provide for those affected by crises.

## Appendix 1 Interview schedule for trainers

### Go through informed consent processes (see separate sheet)

#### Background information

Ask the interviewee for the following information:

- County they were based in during the period of interest
- Whether they worked in a primarily urban or rural setting
- Number of years they have been in practice
- Profession/position

Also record the interviewee's gender.

#### Factual information

1. What previous MHPSS training had you taken part in before you were trained in PFA?
2. What was your role when you were selected for the PFA ToT?
3. Had you ever used PFA yourself before you were trained as a trainer?
4. When did you do the PFA ToT? Who trained you? (organisation) How long was the training?
5. What supervision or support did you receive afterwards, as you began to roll out the training to others?
6. What refresher training did you receive?
7. How many trainings did you deliver? To which groups of people? Over what period of time?

#### Fidelity to the model

1. How long were the training sessions you delivered?
2. What materials did you use to deliver the training?
3. How did you feel about the training materials you used? [Explore what was helpful about them, what was not helpful]
4. Did you have access to the full version of the PFA facilitation manual? [Either hard copy, soft copy, or just the internet link so they could access it]
5. Did you make any modifications to the materials? Did you adapt the materials for use with different types of participants? If so, what adaptations did you make? [For example, different case scenarios for role plays or illustration purposes]

#### Reflections on the ToT model used

1. Why do you think you were selected for the ToT?
2. How did you feel about the ToT? [Probe: what was good about it? What was challenging about it? How confident did you feel to deliver PFA training by the time it finished?]
3. How did you feel about the supervision or support received after the ToT (if any)? Was it helpful? What would have made it more helpful?

*If did not receive*: How did you feel about the lack of supervision? What kind of support would have helped you to feel more confident/comfortable?

1. How did you feel about the refresher trainings you received (if any)? What did they add to the training you had already received?

*If did not receive*: Do you think refresher training would have been helpful? Why? How long after the first ToT should it have taken place?

#### Reflections on the roll-out process

1. Who selected the people who should be trained? Why were these groups/individuals selected? Do you think the selection was done in the right way? Would you select people differently for the training if you did it again? What criteria would you use to select people for PFA training?
2. What do you think about the way your organisation planned to get the PFA training to reach to the people? [What was good about it? What was not so good about it?]
3. What were the good parts of your experience in conducting this training?
  - Which elements of PFA did you find it easy to train people in?

1. How did the people you trained benefit from the PFA training?
2. What challenges did you experience in conducting this training? Were you able to address these challenges?
  - Which elements of PFA did you find it difficult to train people in?

1. What challenges did the participants experience in developing the skills and using them in practice?
2. What do you think about the timing of the training?
  - What were some of the challenges in doing the training during the time of Ebola?

1. Were you able to provide follow-up support to those you trained?
2. If you were going to plan a series of PFA trainings in the future, what would you do differently?
3. If we were going to roll out PFA training in another country similar to Sierra Leone, what advice would you give us?

#### Optional

If there is time, go through ‘fidelity checklist’ with the PFA trainer to find out what they were able to cover and what they were not. Discuss constraints on including certain elements of the training – whether decisions about what to include were based on time, relevance of material or other factors.

#### Close

1. I've asked all the questions I have. Is there anything you would like to add before we close?

Thank the participant for their time, and close the interview.

## Appendix 2 Interview schedule for PFA providers

### Go through INFORMED CONSENT processes (see separate sheet)

#### Background information:

Ask the interviewee for the following information:

- County they were based in during the period of interest
- Whether they worked in a primarily urban or rural setting
- Number of years they have been in practice
- Profession/position

Also, record the interviewee's gender.

#### Factual information

1. What training had you taken part in before you were trained in PFA? [Including basic training]
2. What was your position when you were selected for the PFA training?
3. When did you participate in the PFA training? Who trained you (organisation)? How long was the training?
4. What materials and training methods did they use? Did you get any materials to take away after the training?
5. What supervision and support did you receive as you started to use the PFA approach? [Ask about peer supervision as well]
6. What refresher training did you receive? How long after your initial training did this take place?

#### Reflections on the PFA training

1. What challenges were you experiencing in your work before you did the PFA training? [If any]
2. To what extent did the PFA training help you to address those challenges?
3. Why do you think you were selected for the training?
4. How did you feel about the training workshop?
  - What was good about it? What was challenging about it? What would have made it better?
  - Did you enjoy the training?
  - How did you find the training methods? What was helpful? What was less helpful?
  - What do you think about the timing of the training?
  - How confident did you feel to use the PFA approach by the time the training finished?

1. What would you change about the training, if you were going to recommend for people planning to deliver it in the future?
2. How did you feel about the supervision or support received after the training (if any – including peer supervision)? Was it helpful? What would have made it more helpful?

*If did not receive*: Do you think supervision or support would have been helpful? Why?

1. How did you feel about the refresher trainings you received (if any)? What did they add to the training you had already received?

*If did not receive*: Do you think refresher training would have been helpful? Why? How long after the first training should it have taken place?

#### Reflections on using the PFA skills after the training

1. After the training, have you been using the PFA skills in your work?

*If no* – why not? [Discuss reasons] Have you used the skills outside work?

*If yes* – [ask the questions below]

1. How did PFA fit within your role? In what context were you able to use the PFA skills?
2. After the training, did you find that relationships in your work team changed?
3. How did the training help you? [Explore what specific elements the interviewee experienced as helpful, and how they helped her/him. Get examples of how they were helpful, if possible.]
4. How did the PFA training change your behaviour/the way you worked? [include general orientation, approach as well as using the specific skills].
5. What were the good things about using PFA in your role?
6. What were the challenges you experienced in using PFA?
7. Can you tell us about a time when you used the PFA skills? [Talk them through the example]
  - Can you summarise what happened in that case, and what you did?
  - Do you remember the PFA Action Points? [Make sure they know to prepare, look, listen, link – remind if necessary]
    - Can you tell me what you did to ‘prepare’ in this case?
    - Tell me about how you applied the ‘look’ stage of PFA in this case? (Any challenges?)
    - Tell me about how you applied the ‘listen’ stage of the PFA approach in this case? (What skills did you use? What challenges did you face? How did the person you were helping respond to this stage?)
    - Tell me about how you applied the ‘link’ stage of the PFA approach in this case? (What skills did you use? What challenges did you face? How did the person you were helping respond to this stage?)
  1. If you think about this particular case, what did you find helpful about the PFA approach?
  2. And what parts of the PFA approach did you find unhelpful or unable to use in this case?

1. Over the time that you have been using PFA, did you adapt or change any elements of the approach, to make it better or easier to use? If so, which elements?
2. Did the PFA training help you to care for yourself and your colleagues? Or perhaps for your family or friends? If yes, in what ways?
3. Have you found that PFA was more useful with particular types of people? Which types? Were there any groups who haven't responded well to PFA?
4. Were there any situations in which you thought that providing PFA may be harmful?
5. Can you tell us about the kinds of people you have needed to refer for other types of help, who you haven't been able to help yourself?
6. Were you able to make the referrals you needed to? Were there gaps in the referral pathways? If yes, what were the gaps? How could these have been addressed?
7. Are you still using the PFA skills now? If yes – in what context? If no – why not?

#### Close

1. I've asked all the questions I have. Is there anything you would like to add before we close?

Thank the participant for their time, and close the interview.
