# The myth of the 1-day training: the effectiveness of psychosocial support capacity-building during the Ebola outbreak in West Africa – ADDENDUM

**DOI:** 10.1017/gmh.2019.10

**Published:** 2019-06-17

**Authors:** Rebecca Horn, Fiona O'May, Rebecca Esliker, Wilfred Gwaikolo, Lise Woensdregt, Leontien Ruttenberg, Alastair Ager

**Key words:** Capacity building, Ebola, Liberia, mental health, psychological first aid, psychosocial support, Sierra Leone

The article originally stated that the authors were supported by a grant from ELHRA as one of its R2HC projects. To expand on this, the work reported was funded by ELRHA's Research for Health in Humanitarian Crises (R2HC) Programme, which aims to improve health outcomes by strengthening the evidence base for public health interventions in humanitarian crises. The R2HC programme is funded by the UK Government (DFID), the Wellcome Trust, and the UK National Institute for Health Research (NIHR).
